# Lack of knowledge regarding HPV and its relation to oropharyngeal cancer among medical students

**DOI:** 10.1002/cnr2.1517

**Published:** 2021-07-22

**Authors:** Malik Sallam, Deema Dababseh, Alaa Yaseen, Ayat Al‐Haidar, Hajar Ettarras, Dania Jaafreh, Hanan Hasan, Khaled Al‐Salahat, Esraa Al‐Fraihat, Yazan Hassona, Gülşen Özkaya Şahin, Azmi Mahafzah

**Affiliations:** ^1^ Department of Pathology, Microbiology and Forensic Medicine, School of Medicine The University of Jordan Amman Jordan; ^2^ Department of Clinical Laboratories and Forensic Medicine Jordan University Hospital Amman Jordan; ^3^ Department of Translational Medicine, Faculty of Medicine Lund University Malmö Sweden; ^4^ School of Dentistry the University of Jordan Amman Jordan; ^5^ Department of Basic Medical Sciences, Faculty of Medicine Al‐Balqa Applied University Salt Jordan; ^6^ Department of Oral and Maxillofacial Surgery, Oral Medicine and Periodontology Jordan University Hospital Amman Jordan; ^7^ Department of Clinical Microbiology, Laboratory Medicine Skåne University Hospital Lund Sweden

**Keywords:** carcinoma, medical education, oropharyngeal cancer, sexually transmitted infection

## Abstract

**Background:**

Oropharyngeal cancer (OPC) is an important cause of cancer‐related mortality. Early detection of OPC results in a favorable prognosis and higher survival rates. Infection by high‐risk types of human papillomavirus (HPV) is a risk factor for OPC with an upward trend globally. Medical students' knowledge and awareness of HPV‐related OPC can be crucial in the preventive efforts.

**Aim:**

To assess HPV knowledge among medical students at the University of Jordan, with particular focus on its relation to different cancers.

**Methods:**

This paper‐based survey study was conducted in November 2019. The survey items were based on previously validated surveys used to evaluate HPV‐related OPC knowledge among dental students and professionals. To assess HPV knowledge and students' confidence in personal history taking and physical examination, we developed a knowledge and confidence scores that showed acceptable reliability.

**Results:**

The total number of participants was 1198 students, with a median age of 21 and female predominance (*n* = 697, 58.2%). Among the participants, 93.3% heard of HPV prior to this survey (*n* = 1118). Higher levels of knowledge regarding cervical cancer, OPC and HPV vaccination was seen among clinical students compared to their preclinical counterparts, but their overall HPV knowledge was low. Only 18.4% and 21.0% of the clinical students correctly identified the association of HPV with penile and oropharyngeal cancers, respectively. Additionally, 34.5% of the clinical students were not aware of the availability of HPV vaccines. The majority of students (92.0%) reported that the university courses were their major source of knowledge about HPV.

**Conclusion:**

A profound lack of knowledge regarding HPV role in OPC was found among medical students. This insufficiency included several aspects of the virus and its associated diseases. Such gaps in knowledge could have negative consequences in early detection and prevention of OPC and should be addressed by evaluation of the current curriculum.

## 1 INTRODUCTION

The human papillomavirus (HPV) comprises a group of non‐enveloped DNA viruses that have a tropism for epithelial cells.^1^ More than 100 types of HPV have been identified so far, with a predilection of certain types to cause infection in distinct anatomic sites.^2^ Several HPV types are associated with common warts and condylomas (benign and low risk types, e.g., HPV‐6 and HPV‐11), while others are considered as oncoviruses (high‐risk types, e.g., HPV‐16, HPV‐18 and HPV‐31).^1^, ^3^ These high‐risk types usually cause inapparent infection, which can progress to high‐grade neoplasia and invasive cancer.^1^

The HPV‐related malignancies include cervical, oral/oropharyngeal, anal, vulvovaginal and penile cancers.^4^ At non‐cervical sites, HPV‐related cancers (e.g., HPV‐related oropharyngeal cancer) represent distinct entities from their non‐HPV‐related counterparts, and the adoption of screening and active immunization preventive measures can greatly reduce the burden of such cancers and its precursors.^4^, ^5^, ^6^

There is no specific curative treatment for HPV.^7^ However, effective and safe vaccination to prevent infection by the most common high‐risk types; namely, HPV‐16 and HPV‐18 has been available for more than a decade. HPV vaccination is recommended for both sexes a few years prior to sexual debut.^5^, ^8^ Specifically, the latest recommendations by the Centers for Disease Control and Prevention (CDC) stated that ‘HPV vaccination is routinely recommended at age 11 or 12 years’.^9^ Additionally, the CDC recommended catch‐up vaccination for all persons through 26 years of age who are not adequately vaccinated.^9^ Despite being approved for adults aged 27 through 45 years, HPV vaccination is not currently recommended for this age group.^9^ Moreover, important steps have been conducted in the pursue of achieving therapeutic vaccines (aiming to clear established infections) against HPV‐related cancers; nevertheless, more studies are needed to elucidate the full potential of such a strategy.^10^, ^11^

Screening for early HPV‐induced premalignant changes in cervical cells can be done using the Papanicolaou smear (Pap smear), which is considered an effective cancer preventive tool.^12^ An equivalent procedure to Pap smear is currently lacking for screening of oropharyngeal cancers. Nevertheless, Pap smears cannot detect all HPV‐related changes.^13^ Hence, robust preventive measures also rely largely on vaccination.

Additionally, HPV is among the most common causative agents of sexually transmitted infections (STIs), and it can be acquired by non‐sexual routes including direct contact and the indirect route via fomites.^14^, ^15^ For oral HPV infections, the risk factors for acquisition include open‐mouth kissing, in addition to the life‐time number of oral sexual partners.^16^, ^17^, ^18^ Other studies displayed the presence of other risk factors for oral HPV infections including the number of sexual partners, alcohol use and smoking.^17^, ^19^

Medical students constitute a significant group to be targeted with focused knowledge on specific subjects, including the screening and identification of HPV‐related premalignant lesions.^20^, ^21^ As future physicians, medical students must be aware of the association between HPV and cancer (e.g., cervical and oropharyngeal cancer), in addition to the need for improved knowledge about HPV.^22^, ^23^ Consequently, this knowledge could result in a significant impact on patients' survival through the detection of the premalignant lesions. Knowledge and awareness about HPV could also motivate the recommendation of HPV vaccination.^20^, ^22^ Currently, HPV vaccination has not been part of the national immunization program in Jordan, similar to a majority of Arab countries.^24^, ^25^

In our previous work that involved a survey among dental students at the University of Jordan (UJ), the knowledge and awareness levels regarding various aspects of HPV‐related oropharyngeal cancer were satisfactory.^26^ As a follow‐up project, we aimed to assess the knowledge of medical students at UJ regarding different aspects of HPV infection. The main focus of this study was to evaluate the medical students' awareness that HPV can cause oropharyngeal cancer and their awareness that effective HPV vaccines do exist. This subject is of particular importance considering the sexual connotations associated with HPV and the social barriers that can preclude student training in such subjects. The social and psychological consequences of HPV infections can result in stigma and anxiety, with concerns involving sexual relationships.^27^

## 2 METHODS

### 2.1 Study design

This cross‐sectional study was conducted using a paper‐based survey. This survey was partly based on our previous study that was used to assess UJ dental students' awareness and attitudes towards HPV‐related oropharyngeal cancer.^26^ The development phase of the questionnaire was based on survey items of validated questionnaires from USA, Netherlands and Spain, which aimed to assess HPV‐related oral cancer knowledge and attitude among dental students and professionals.^28^, ^29^, ^30^ At the time of survey distribution, medical education at UJ comprised six years with the first year being a pre‐med year having both medical and dental students. Hence, first‐year students were not included in this study. Survey distribution among medical students at the School of Medicine/UJ was done in‐person following classroom lectures. It started on 19 November 2019 and concluded on 28 November 2019. For each curriculum year, survey distribution took a maximum of two days. The number of target students at each curriculum year was as follows: 2nd Year = 647, 3rd Year = 435, 4th Year = 389, 5th Year = 361 and 6th Year = 380 students. The study population was divided into two categories: ‘pre‐clinical’ students involving 2nd and 3rd year students, and ‘clinical’ students involving 4th, 5th and 6th years students.

Since English is the official teaching language at the Medical School/UJ, the questionnaire was developed and distributed in English.

### 2.2 Ethical permission

The study was approved by the Deanship of Scientific Research at UJ and by the Institutional Review Board (IRB) at Jordan University Hospital (JUH) on 21/10/2019 (decision No. 223/2019, reference No. 10/2019/23154). The study was conducted anonymously, and all data were treated with confidentiality. To keep anonymity of the students in this study, verbal consent was obtained from the participants as approved by JUH IRB.

### 2.3 Survey items

The survey comprised 21 items as follows: a section on demographic characteristics of each participant (age, gender, nationality, curriculum year and grade point average [GPA]), a defining question on whether the participant has heard of HPV before the survey. For those who answered (Yes) to the previous question, a request to complete the survey followed with a 6‐item section on HPV knowledge, involving (Yes/No/I do not know) questions, and a 9‐item section on the transmission routes and HPV‐associated clinical disease (including HPV association with cervical, oral and penile cancers), and an item to assess the main source of knowledge about HPV. Finally, a section was included using a 5‐point Likert scale to assess how comfortable does the participant feel during history taking that involves discussing personal topics with patients and attitude towards physical examination.

### 2.4 HPV knowledge score calculation

The content validity of the current questionnaire involving relevance, and representativeness was conducted by three authors (MS, YH and AM) who are involved in medical and dental education at UJ. To assess the overall knowledge of the participants regarding HPV, we used an HPV Knowledge Score (HPV K‐score), consisting of the following 15 points: 1) ubiquity of HPV infections; 2) existence of more than 100 HPV types; 3) only a group of HPV types are associated with cancer; 4) inapparent nature of most HPV infections; 5) existence of HPV vaccines; 6) failure of Pap smears to detect all HPV‐induced premalignant lesions; 7) HPV association with cervical cancer; 8) HPV association with oropharyngeal cancer; 9) HPV association with penile cancer; 10) lack of HPV association with nasopharyngeal cancer; 11) lack of HPV transmission through blood transfusion; 12) sexual transmission of HPV; 13) cold sores are not caused by HPV; 14) genital warts are caused by HPV; 15) common warts are caused by HPV. Each correct response for any of the previous points was scored as a single point, while incorrect response/non‐response (implying lack of knowledge) or I don't know response (for the first six points) was scored as zero. Thus; the maximum possible HPV K‐score was 15 while the minimum was zero. The Cronbach's alpha value of 0.737 ensured an acceptable internal consistency for the proposed HPV K‐score.

### 2.5 Confidence scale calculation

The evaluation of clinical students' attitude towards history taking that involves discussing personal topics with the patients and towards physical examination was done using personal history taking and physical examination confidence scale (Confidence scale). The participants were asked to report how comfortable they feel with each of the following five points: 1) asking patients questions regarding their lifestyle; 2) asking patients about sexually transmitted infections; 3) asking patients about sexual abuse; 4) physically examining the patient; 5) physically examining a patient of the opposite sex. The scale ranged from ‘not comfortable at all’, which was given a minimum score of 1, to ‘most comfortable’, which was given a maximum score of 5. Higher Confidence scale values indicated greater comfort in attitude towards discussing personal topics with patients and towards physical examination. The Cronbach's alpha value of 0.774 ensured an acceptable internal consistency for the proposed Confidence scale.

### 2.6 Statistical analysis

All statistical analyses and data description were done using IBM Statistical Package for the Social Sciences (SPSS) 22. Statistical significance was considered for *p* < 0.050. For scale variables (age, HPV K‐score and Confidence scale), mean and standard deviation (SD) were calculated. Chi‐squared test (*χ* ^2^ test) was used to assess the statistical difference between categorical variables while Mann–Whitney *U* (M–W) and Kruskal–Wallis (K–W) tests were used to assess the statistical difference between categorical and scale variables. Univariate analysis of variance was used to assess the potential effect of confounding variables (sex, nationality, curriculum year and GPA) in relation to Knowledge and Confidence scores.

## 3 RESULTS

### 3.1 Study population

The total number of the study participants who completed at least one item of the questionnaire was 1198 out of 2212 medical students at the School of Medicine/UJ during the time of survey distribution. For gender distribution, females comprised 58.2% (*n* = 697) of the study participants and the median age for participants was 21 years (mean = 20.7, interquartile range = 19–22). Jordanian students predominated the study population (*n* = 866, 72.3%), while students of non‐Jordanian citizenship formed 27.6% of all participants (*n* = 331). Students of non‐Jordanian citizenship were distributed among 23 different nationalities with Kuwait, Iraq, Palestine and Syria forming the most common countries of origin (*n* = 232). The highest response rate was found among the second‐year students (*n* = 406, 62.8%), while the lowest was for the sixth‐year students (*n* = 167, 43.9%). Students with GPA higher than 3.0 (very good and excellent) formed 73.4% of all participants who stated their GPAs (Table 1).

**TABLE 1:** Characteristics of the study participants

Characteristic		Number	Percentage
Age^a^ Mean, (SD^b^)		20.7 (1.7)	
Sex	Male	501	41.8%
	Female	697	58.2%
Nationality	Jordanian	866	72.3%
	Non‐Jordanian^d^	331	27.6%
	Item non‐response	1	0.1%
Curriculum year	2nd Year	406	33.9%
	3rd Year	253	21.1%
	4th Year	192	16.0%
	5th Year	180	15.0%
	6th Year	167	13.9%
Level of study	Pre‐clinical	659	55.0%
	Clinical	539	45.0%
GPA^c^	Less than 2.0	5	0.4%
	2.00–2.49	63	5.3%
	2.50–2.99	234	19.5%
	3.00–3.49	392	32.7%
	3.50–4.00	483	40.3%
	Item non‐response	21	1.8%

^a^ Age: With missing data from 44 participants.

^b^ SD: Standard deviation.

^c^ GPA: Grade point average.

^d^ Non‐Jordanian: Countries of citizenship that comprised 23 different countries, with the most common being Kuwait, Iraq, Palestine and Syria.

### 3.2 Knowledge of HPV existence among the study participants

**FIGURE 1:**
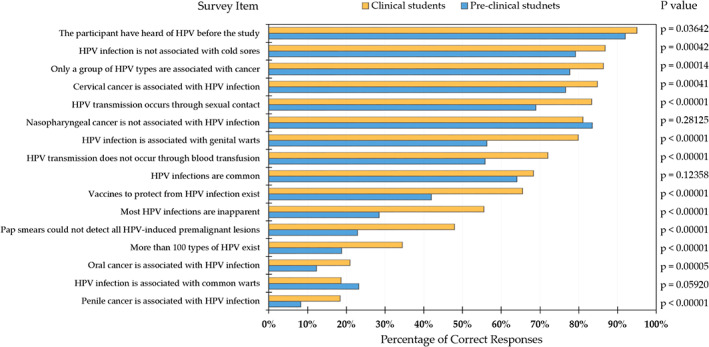
Human papilloma virus (HPV) knowledge among the study participants divided by the level of study (pre‐clinical vs. clinical students). The percentage of correct responses for each one of the fifteen items used to assess HPV knowledge besides the item used to assess whether the participants have heard of HPV before the study. P‐values were calculated using the chi‐squared test

Overall, 93.3% of all participants stated they heard of HPV, with 80 students who stated that they have not heard of HPV before the study. Non‐Jordanian students were less likely to have heard about HPV compared to their Jordanian counterparts (90.3% vs. 94.6%, *p* = 0.008, *χ* ^2^ test). Also, clinical students were more likely to have heard of HPV compared to pre‐clinical students (95.0% vs. 92.0%; *p* = 0.036, *χ* ^2^ test, Figure 1). An incremental increase in the proportion of students who heard of HPV was noticed with the increase in GPA from 80.0% among those with GPA < 2.0, 84.1% among those with GPA of 2.0–2.5, 89.7% among those with GPA of 2.5–3.0, 93.4% among those with GPA of 3.0–3.5, reaching 96.5% among those with GPA > 3.5.

### 3.3 Assessment of HPV knowledge per item among the study participants

Out of the fifteen items used to assess HPV knowledge among the participants, the clinical students displayed a statistically higher percentage of correct responses compared to the preclinical students for twelve items. For the remaining three items, no statistically significant differences were found (Figure 1).

Notably, only 18.4% and 21.0% of the clinical students correctly identified the association of HPV with penile cancer and oropharyngeal cancer, respectively. Additionally, 34.5% of the clinical students were not aware of the existence of HPV vaccines. Correct identification of HPV association with cervical cancer was found among 84.8% of the clinical students (Figure 1).

Comparisons of HPV knowledge per item in relation to sex and nationality among the whole study participants, showed that females had higher knowledge of the existence of HPV vaccines compared to males (55.1% vs. 49.1%; *p* = 0.041, *χ* ^2^ test). Males showed a higher knowledge that Pap smears may not detect all HPV‐induced cervical premalignant changes (37.7% vs. 31.6%; *p* = 0.027, *χ* ^2^ test). Also, males showed higher knowledge of HPV association with oral and penile cancers compared to females (19.2% vs. 14.1%; *p* = 0.018 and 17.0% vs. 9.9%; *p* < 0.001, *χ* ^2^ test). Correct identification that HPV cannot be transmitted through blood transfusion was seen more frequently among females (66.7% vs. 58.1%; *p* = 0.002, *χ* ^2^ test). For non‐Jordanian students, lower level of knowledge regarding HPV association with cervical cancer was seen compared to Jordanian students (73.1% vs. 83.1%; *p* < 0.001, *χ* ^2^ test). Also, non‐Jordanian students had a lower knowledge about sexual transmission of HPV compared to Jordanian students (70.4% vs. 77.4%; *p* = 0.012, *χ* ^2^ test).

### 3.4 Assessment of the overall HPV K‐score

The HPV K‐score was used to evaluate the overall knowledge per curriculum year, sex and nationality. The main improvement in HPV knowledge was seen moving from the fourth curriculum year (mean = 7.3, SD = 3.2) into the fifth curriculum year (mean = 9.8, SD = 2.2; *p* < 0.001, M–W). The overall HPV knowledge which was deduced through HPV K‐score, was higher among Jordanian students (mean = 8.2, SD = 2.9) compared to their non‐Jordanian counterparts (mean = 7.6, SD = 3.2; *p* = 0.005, M–W).

Univariate analysis with HPV K‐score as the dependent variable, curriculum year as the fixed factor, and the following as covariates: sex; nationality; and the latest GPA, showed that the improvement in HPV knowledge was associated with progression through curriculum years (*p* < 0.001). Among the covariates, the latest GPA was found as a significant predictor of HPV K‐score (*p* < 0.001).

### 3.5 Assessment of the confidence scale and attitude towards history taking and physical examination

The total number of the clinical students with a valid confidence scale was 509/539 (94.4%). Upon comparing the Confidence scale among the different study variables (sex, nationality, GPA, different HPV K‐score categories), we did not find any statistically significant differences. For clinical students, no significant improvement in the Confidence scale was found moving from 4th curriculum year (mean = 14.8, SD = 4.3), to 5th curriculum year (mean = 14.1, SD = 3.9) and finally among 6th curriculum year students (mean = 14.9, SD = 4.5; *p* = 0.235, K–W).

For each confidence item, the lowest level of comfort was noticed for history taking involving asking the patients about sexual abuse (mean = 1.9, SD = 1.2), followed by history taking that involves asking the patients about history of STI. For the five items, no statistically significant differences were found in relation to gender, nationality and curriculum year except for asking the patients about their lifestyle (Table 2).

**TABLE 2:** Confidence of the clinical students in personal history taking and physical examination

Confidence scale item	How comfortable are you when asking the patients questions regarding their lifestyle?	How comfortable are you when asking the patients about sexually transmitted infections?	How comfortable are you when asking the patients about sexual abuse?	How comfortable are you when physically examining the patients?	How comfortable are you when examining a patient of the opposite sex?
Overall mean (SD)^a^	3.9 (1.1)	2.4 (1.2)	1.9 (1.2)	3.4 (1.3)	3.0 (1.3)
*Sex*					
Male	3.8 (1.1)	2.5 (1.2)	2.0 (1.2)	3.5 (1.2)	2.9 (1.3)
Female	3.9 (1.1)	2.3 (1.2)	1.9 (1.2)	3.3 (1.3)	3.0 (1.3)
*P*‐value^b^	0.280	0.061	0.190	0.185	0.409
*Nationality*					
Jordanian	3.9 (1.1)	2.4 (1.2)	1.9 (1.2)	3.4 (1.3)	3.0 (1.3)
Non‐Jordanian	3.8 (1.1)	2.4 (1.2)	1.9 (1.2)	3.5 (1.3)	2.9 (1.4)
*P*‐value^b^	0.515	0.683	0.404	0.552	0.522
*Curriculum year*					
4th year	4.0 (1.2)	2.3 (1.2)	1.9 (1.2)	3.5 (1.2)	3.2 (1.3)
5th year	3.7 (1.2)	2.4 (1.1)	1.9 (1.1)	3.3 (1.3)	2.8 (1.3)
6th year	4.0 (1.0)	2.5 (1.3)	1.9 (1.2)	3.3 (1.3)	3.0 (1.3)
*P*‐value^c^	0.008	0.197	0.794	0.315	0.062

^a^ SD: Standard deviation.

^b^ *P*‐value was calculated using Mann–Whitney *U* test.

^c^ *P*‐value was calculated using Kruskal Wallis test.

Univariate analysis with the confidence scale as the dependent variable, curriculum year as the fixed factor, and the following as covariates: sex; nationality; and the latest GPA, showed the lack of improvement in students' confidence in history taking that involves discussing personal topics with the patients and towards physical examination with progression through curriculum clinical years (*p* = 0.570), with no significant results for the covariates.

### 3.6 Sources of knowledge about HPV

The vast majority of the study participants reported that the university courses were their major source of knowledge about HPV (*n* = 1025, 92.0%), followed by the internet/social media outlets (*n* = 131, 11.8%), and school courses (*n* = 53, 4.8%). The participants who relied on university courses as the source of knowledge about HPV had a higher HPV K‐score compared to those who did not (mean = 8.7, SD = 2.5 vs. mean = 7.8, SD = 2.0; *p* < 0.001, M‐W).

## 4 DISCUSSION

The relevance of this study is related to the following points: First, the study was aimed to evaluate knowledge of medical students at UJ ‐the majority of whom were Arabs‐ towards HPV, which is the most common cause of STIs. In the majority of Arab countries, discussion about STIs is still considered as a taboo subject, which can lead to stigmatization.^31^ Infections by HPV and its related STI can be considered as a sensitive topic in Jordan among other Arab countries, that could be difficult to discuss even among healthcare providers. This could negatively impact their potential recommendation of HPV vaccination.^32^ In addition, medical education is a dynamic process, mandating continuous evaluation to adapt to new updates in literature. Thus, continuous assessment of the taught subjects in medical schools is mandated. Moreover, evaluation of medical students' and doctors' knowledge about HPV‐related oropharyngeal cancer is important since it is more likely for the patients having oral lesions to seek help from medical doctors rather than dentists. Hence, it is important to assess the suitability of training for the future medical doctors.^33^

The results of this study highlighted the presence of major gaps in HPV knowledge among medical students at UJ. Despite the significant improvements in the level of knowledge shown upon advancing through curriculum years, the overall level of knowledge ‐especially among clinical students‐ was worrisome. For example, less than a quarter of the clinical students knew about HPV association with oropharyngeal cancer, and less than one‐fifth were aware of HPV association with penile cancer. Lack of HPV knowledge in addition to unawareness about the link of HPV with oral/oropharyngeal cancer was seen in recent survey studies with suggested interventional measures including interactive workshops for preclinical students and team‐based learning.^20^, ^21^, ^34^, ^35^ A recent study that surveyed medical and non‐medical students from Ukraine showed a poor level of knowledge regarding HPV‐related oral and rectal cancers among all groups of students.^36^ Another recent study that surveyed female college students from China, reported similar defect in knowledge about the ability of HPV to cause oral cancer.^37^

A recent systematic review by Parsel et al. illustrated the presence of the knowledge gap of HPV‐associated oropharyngeal cancer among health‐care providers.^38^ This review emphasized the need and importance of additional education aiming to increase the awareness of HPV‐related oropharyngeal cancer, which can have important results in the prevention and earlier detection of the disease.^38^

Another important defect in HPV knowledge detected among medical students in this study was the relatively high percentage of unawareness regarding the presence of effective HPV vaccines. This was also seen in a recent report among Brazilian medical students by Costa et al.^39^ Only 42.0% of preclinical students in this study were aware of the availability of HPV vaccines compared to 65.5% of the clinical students. Vaccination program for HPV in Jordan has not been present so far, despite the high prevalence of HPV in a few studies conducted in Jordan.^25^, ^40^ Thus, well‐designed training sessions and programs besides awareness campaigns are needed to address this gap of knowledge regarding HPV vaccines not only among medical students, but also among other college students and the general public in Jordan. Despite the availability of HPV vaccination in Jordan for individuals who are willing to pay, more studies are needed to assess the potential benefit of its inclusion in the national vaccination program. This entails nationwide studies investigating the prevalence of HPV among patients with STIs and cervical cancer in the country.

An important result in this study was the huge discrepancy in awareness of HPV association with oral cancer between medical students and dental students at UJ. In our previous work among dental students at UJ, 88.2% of the clinical students correctly identified HPV as a risk factor for oropharyngeal cancer compared to 21.0% among medical students in this study.^26^ This difference was previously reported by Awan et al. among medical and dental students in Malaysia.^41^ Possible explanation for this result can be related to more focused courses on topics like oral microbiology and oral pathology for Doctor of Dental Surgery Students at UJ. Thus, the current approach of teaching this subject among medical students should be revised in light of relative success in the outcome among dental students.

In this study, female students displayed a higher level of knowledge regarding HPV vaccination, whereas male students were more aware of HPV association with oral and penile cancers. This can be explained by the importance of HPV vaccination in the prevention of cervical cancer, which is a leading cause of morbidity and mortality among females. On the contrary, the role of HPV vaccination in the prevention of oropharyngeal cancers is yet to be fully appreciated.

For the clinical students, we did not observe a significant improvement in self‐reported comfort in history taking tackling sensitive topics and in attitude towards physical examination. This result should be considered in the clinical training particularly for aspects of history taking involving asking about STIs and history of possible sexual abuse. This result points to the importance of refining the current teaching methods to improve the interpersonal and communication skills. This in turn can help to boost the students' confidence in history taking that involves discussing personal topics with the patients.^42^

Since the University courses were the most common reported source of HPV knowledge by a mile, the content, approach and teaching style should be improved. This would help greatly in reducing the burden of HPV‐related diseases. Further research is strongly recommended to assess the required theoretic and practical teaching strategies that would help to prepare medical students to be fully aware of HPV‐related cancers and its prevention.^42^, ^43^

### 4.1 Strengths and limitations

The main strength point in this study was related to the large sample size with a coverage rate of 54% of all students at the School of Medicine/UJ. In addition, the validity of HPV K‐score and the Confidence scale supports the conclusions made regarding HPV knowledge and confidence in history taking and physical examination. For the limitations of this study, the results can give a general idea regarding the level of medical students' knowledge on HPV‐related oropharyngeal cancer in Jordan. However, this study might not be representative of the overall medical students' knowledge in Jordan given the presence of an additional five medical schools in the country. The absence of an item in the survey to assess HPV vaccination status among the study participants is an additional limitation. However, the lack of HPV vaccination in Jordan and most Arab countries can be used to make the assumption that a majority of participants have not received the vaccine yet.^25^ Furthermore, a potential caveat that should be considered is sampling error that is related to the decision of the student to participate in the survey. This decision might be influenced by previous knowledge on the study topic, which could result in biased estimation of the level of knowledge among the participants.

## 5 CONCLUSIONS

The main results of this study showed severe lack of knowledge regarding HPV association with oropharyngeal and penile cancers among medical students. Despite the acquisition of HPV knowledge through advancing medical school curriculum years, the overall level of HPV knowledge was unsatisfactory. This included an appreciable proportion of unawareness towards the presence of HPV vaccination. Interventional measures including revising the current microbiology, dermatology and gynecology curricula should be considered. Another approach like team‐based learning is also recommended. Future research following the implementation of revised curricula is strongly recommended to evaluate the potential improvement in students' knowledge and skills.

## CONFLICT OF INTEREST

The authors have stated explicitly that there are no conflicts of interest in connection with this article.

## AUTHOR CONTRIBUTIONS

All authors had full access to the data in the study and take responsibility for the integrity of the data and the accuracy of the data analysis. Conceptualization, Malik Sallam, Gülşen Özkaya Şahin, and Azmi Mahafzah; Methodology, Malik Sallam; Investigation, Malik Sallam, Deema Dababseh, Alaa Yaseen, Ayat Al‐Haidar, Hajar Ettarras, Dania Jaafreh, Hanan Hasan, Khaled Al‐Salahat, Esraa Al‐Fraihat, Yazan Hassona, Gülşen Özkaya Şahin, and Azmi Mahafzah; Formal Analysis, Malik Sallam; Resources, Malik Sallam; Writing–Original Draft, Malik Sallam; Writing–Review & Editing, Malik Sallam, Deema Dababseh, Alaa Yaseen, Ayat Al‐Haidar, Hajar Ettarras, Dania Jaafreh, Hanan Hasan, Khaled Al‐Salahat, Esraa Al‐Fraihat, Yazan Hassona, Gülşen Özkaya Şahin, and Azmi Mahafzah; Visualization, Malik Sallam; Supervision, Malik Sallam, Gülşen Özkaya Şahin, and Azmi Mahafzah; Funding Acquisition, the authors received no financial support for this research.

## ETHICAL STATEMENT

The study was approved by the Institutional Review Board (IRB) at Jordan University Hospital (JUH) on 21/10/2019 (decision No. 223/2019, reference No. 10/2019/23154). To keep anonymity of the students, verbal consent was obtained. This approach was approved by JUH institutional review board.

## Data Availability

The data presented in this study are available on request from the corresponding author (Malik Sallam).
